# Supplementary material for: Differences in 5'untranslated regions highlight the importance of translational regulation of dosage sensitive genes
Source: Genome Biol. 2024 Apr 29;25:111. doi: 10.1186/s13059-024-03248-0 (PMC11057154; doi:10.1186/s13059-024-03248-0)
Supplement: Supplementary file 2 — Additional file 2: Supplementary Tables. [file 13059_2024_3248_MOESM2_ESM.pdf]

Differences in 5'untranslated regions highlight the importance of translational regulation of dosage sensitive genes

Supplementary Figures

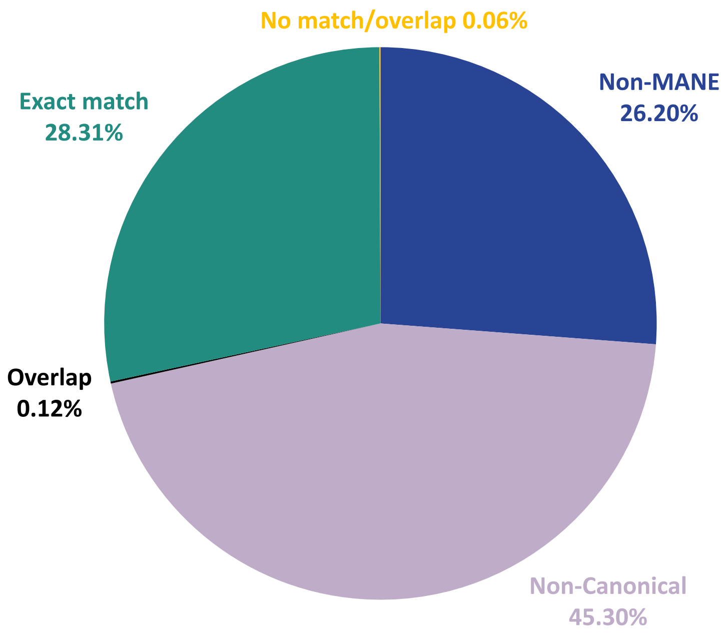

Figure S1: *Overlap between Ribo-seq uORFs (n=5,052) and predicted MANE uORFs (n=18,064). 28.3% of Ribo-seq uORFs matched a predicted uORF exactly. A further 0.12% overlapped with a predicted uORF. 26.2% of Ribo-seq uORFs did not map to MANE transcript 5'UTRs so we did not include in our analyses.45.3% of Ribo-seq uORFs start with a non-canonical start codon (non-AUG) and hence would not be predicted to overlap with the predicted uORF set.*

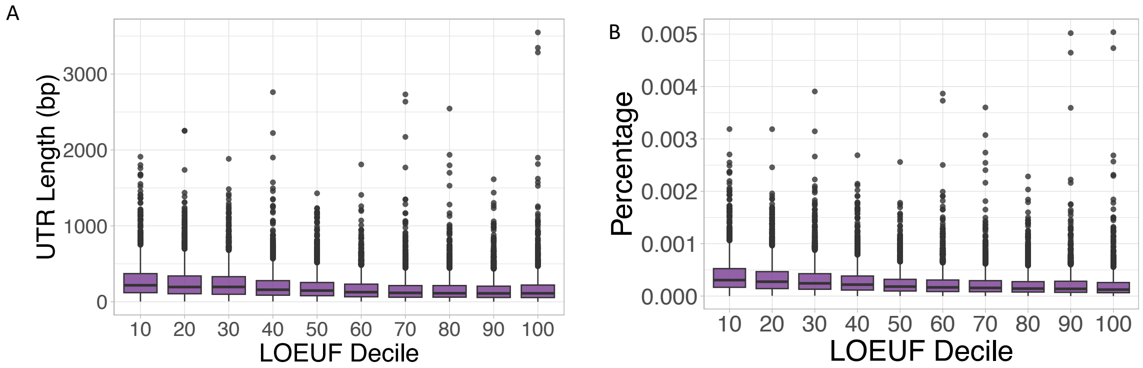

Figure S2: ***A****) 5'UTR length by LOEUF decile with genes within bottom 10% of coding sequence length distribution removed.* ***B****) 5'UTR as a proportion of total mRNA length; 5'UTRs are a significantly larger proportion of the total mRNA in genes most intolerant to LoF (Wilcoxon* $P<1x10^{-15}$*).*

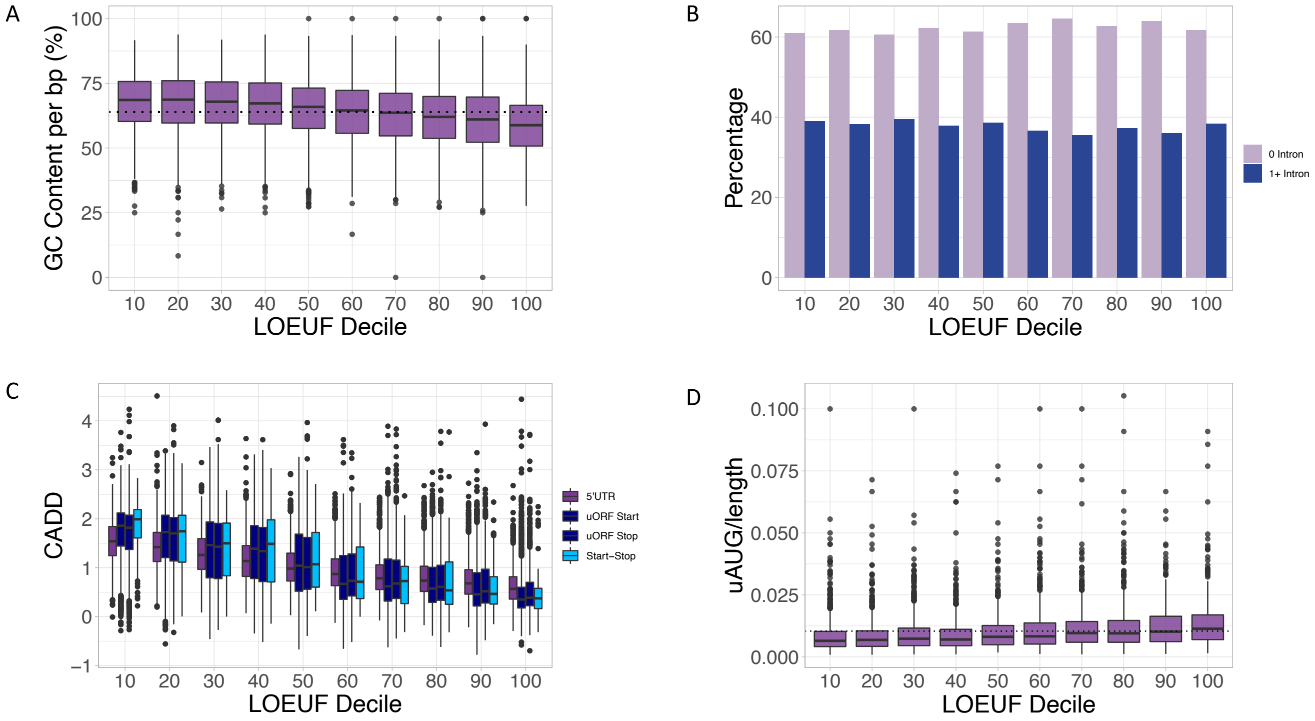

Figure S3: ***A****) Genes most intolerant to LoF had higher GC content than LoF tolerant genes (Wilcoxon P<1x10^{-15}). Average GC content for all genes is 63.9% and is shown by a dotted line.* ***B****) There was no significant difference between 5'UTR intron proportions across LEOUF deciles (Chi-square P=0.19).* ***C****) CADD scores for 5'UTRs, uORFs and start-stops across LEOUF deciles were higher in lower deciles; in genes more intolerant to LoF (T-test 5'UTRs P<1x10^{-15}, uORF start P<1x10^{-15}, uORF stop P<1x10^{-15}, start-stop P<1x10^{-15}).* ***D****) Genes most intolerant to LoF had fewer uAUGs per base pair (Chi-squareP<1x10^{-15}). Average uAUG/length is 0.01 (dotted line). All statistical tests compare the bottom two and top two deciles of LOEUF.*

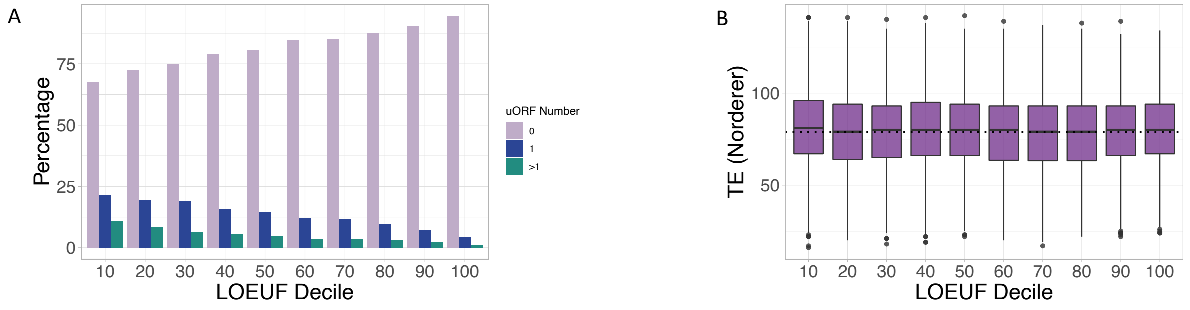

Figure S4: ***A****) The 5'UTRs of genes that are more intolerant to LoF more often contain Ribo-seq uORFs than genes that are tolerant to LoF (Chi-square P<1x10*$^{-15}$*).* ***B****) There was no difference between translational efficiencies (TE) for predicted uAUGs across LEOUF deciles (Wilcoxon P=0.6). Statistical tests compare the bottom two and top two deciles of LOEUF.*

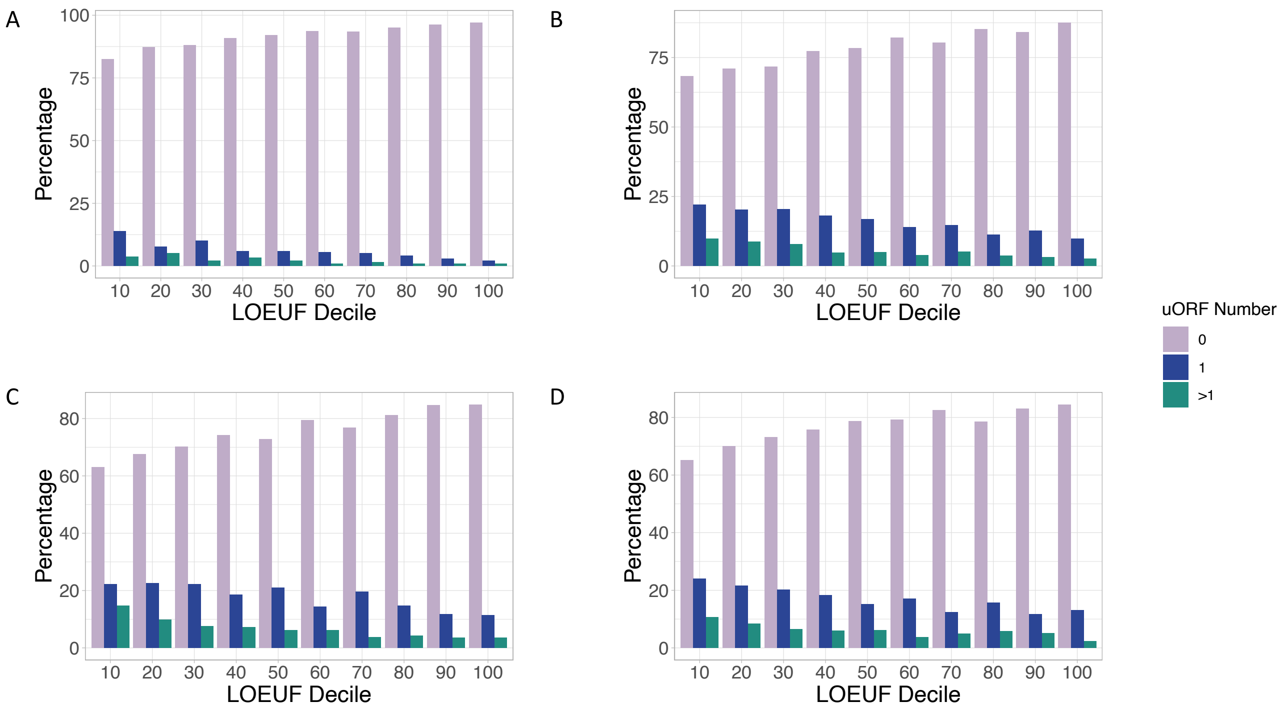

Figure S5: *The 5'UTRs of genes that are more intolerant to LoF more often contain Ribo-seq uORFs than genes that are tolerant to LoF. This remains true after splitting genes into four quartiles based on their expression levels in GTEx, with quartile 1 (Q1) being the lowest gene expression to quartile 4 (Q4) being the highest gene expression level (Chi-square,* ***A****: Q1 P<1x10*$^{-15}$*;* ***B****: Q2 P=3.8x10*$^{-12}$*;* ***C****: Q3 P=6.5x10*$^{-14}$*;* ***D****: Q4 P=8x10*$^{-11}$*).*

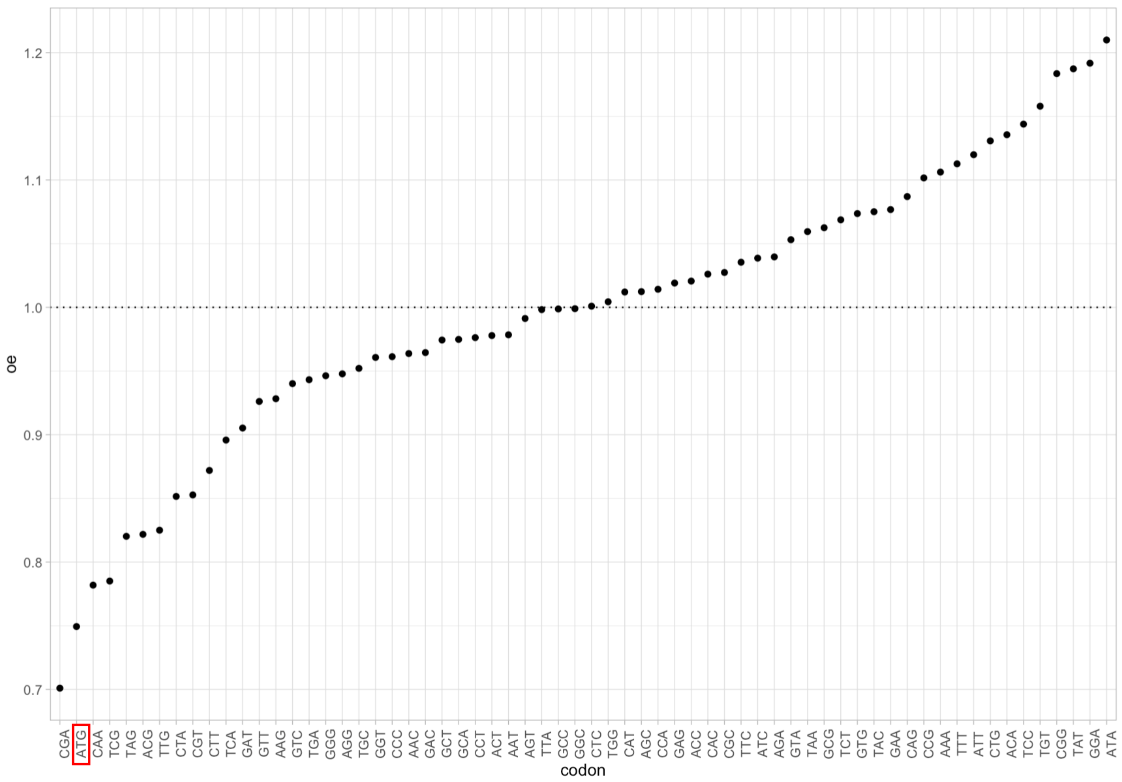

Figure S6: *MANE 5'UTR sequences were shuffled 1000 times to generate an observed/expected (o/e) ratio for all codons in 5'UTRs. o/e values below 1 indicate codon appearing at a lower frequency than expected by chance; exceeding 1 suggests higher than expected frequency. ATG codons were significantly more depleted than would be expected by chance.*

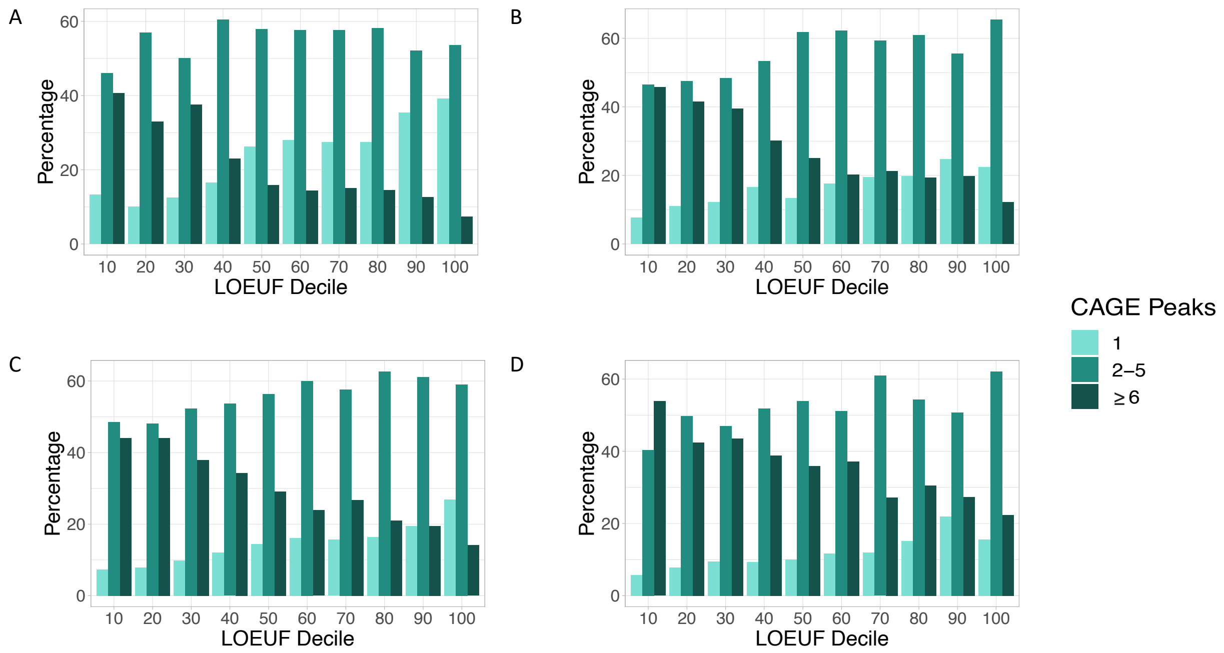

Figure S7: *Genes most intolerant to LoF were significantly more likely to have multiple associated CAGE peaks when compared to genes most tolerant to LoF. This remains true after splitting genes into four quartiles based on their expression levels in GTEx, with quartile 1 (Q1) being the lowest gene expression to quartile 4 (Q4) being the highest gene expression level (All Chi-square,* ***A****: Q1 >1 cage peak $P<1x10^{-15}$ ≥6 cage peak $P<1x10^{-15}$;* ***B****: Q2 >1 cage peak $P=3.8x10^{-12}$ ≥6 cage peak $P<1x10^{-15}$;* ***C****: >1 cage peak Q3 $P=6.5x10^{-14}$ ≥6 cage peak $P<1x10^{-15}$;* ***D****: Q4 >1 cage peak $P=8x10^{-11}$ ≥6 cage peak $P<1x10^{-15}$).*

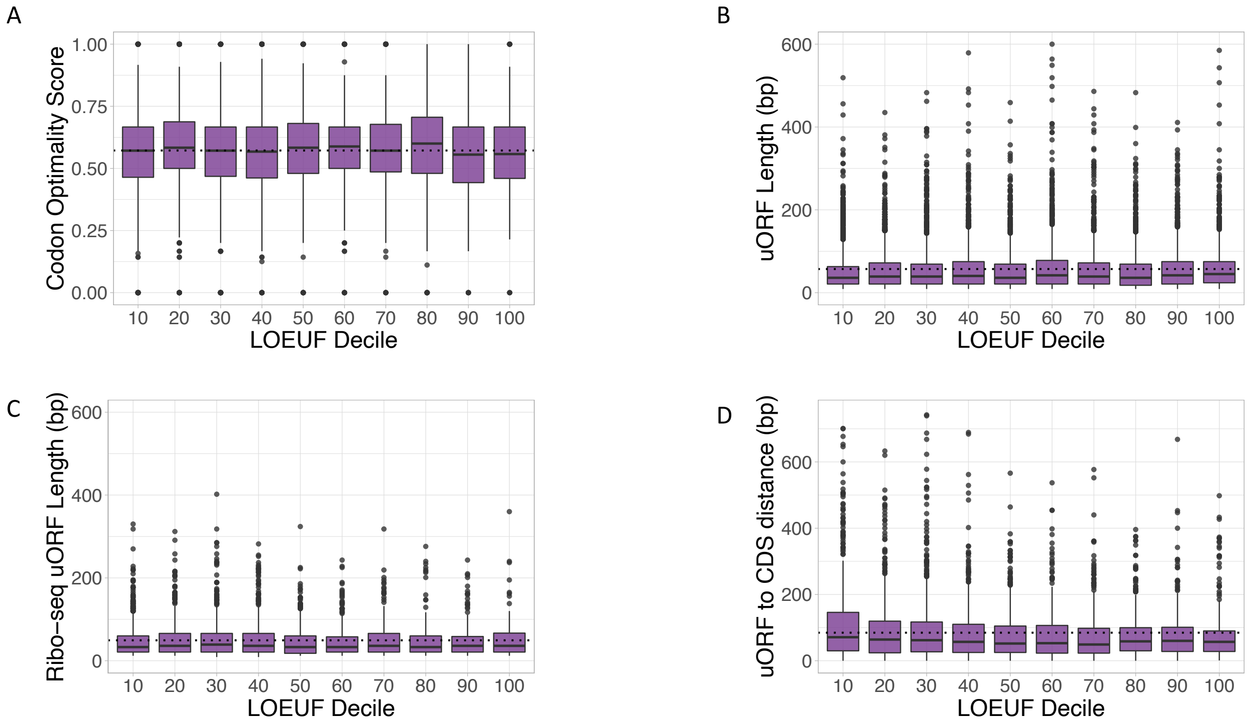

Figure S8: *A) There was no significant difference in codon optimality score for Ribo-seq uORFs between lowest and highest two deciles (Wilcoxon P=0.17), average for all genes is dotted line.* ***B****) Predicted uORFs in the lower two deciles were slightly shorter compared to the highest two deciles (Wilcoxon* $P=4.9 \times 10^{-06}$*). Mean uORF length is shown as a dotted line. The y-axis is truncated at 600bp (7 uORFs were >600bps).* ***C****) There is no significant difference in Ribo-seq uORF lengths between lowest and highest two LOEUF deciles(Wilcoxon, P=0.9). Mean uORF length is shown as a dotted line. The y-axis is truncated at 600bps (1 uORF was >600bps).* ***D****) The distance between the stop codon of the last uORF to the CDS start (of the predicted uORF set) was greater in the genes most intolerant to LoF (lowest versus top two deciles, Wilcoxon* $P=1.3 \times 10^{-04}$*). Average distance is 84.7bps (dotted line). The y-axis is truncated at 750bp (3 genes had distance >750bps).*

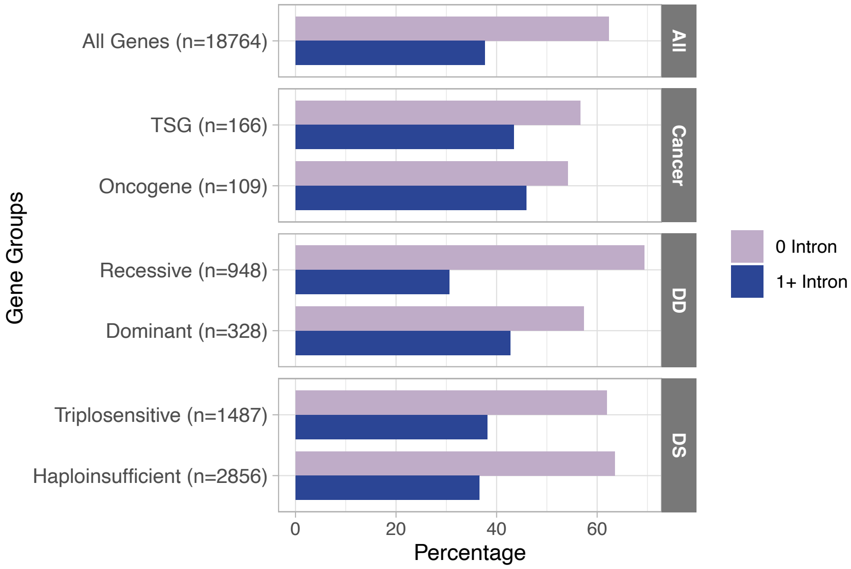

Figure S9: *We did not observe any difference in the proportion of 5UTRs that have introns between disease gene sets and all genes (DD dominant: Chi-square P=0.07; Onc Chi-square P=0.09; TSG Chi-square P=0.15; HS Chi-square P=0.18; TS Chi-square P=0.39), except for DD recessive genes, where significantly fewer had introns (Chi-square P=4.7x10^{-06}).*
