# Supplementary material for: Differences in 5'untranslated regions highlight the importance of translational regulation of dosage sensitive genes
Source: Genome Biol. 2024 Apr 29;25:111. doi: 10.1186/s13059-024-03248-0 (PMC11057154; doi:10.1186/s13059-024-03248-0)
Supplement: Supplementary file 3 — Additional file 3. Peer review history. [file 13059_2024_3248_MOESM3_ESM.docx]

**Review history**

**First round of review**

**Reviewer 1**

mRNA regions upstream of protein coding sequences often contain regulatory elements such as short translated sequences termed uORFs and RNA secondary structures that interfere and alter progression of ribosomal scanning complexes thus influencing efficiency of translation initiation of proteins encoded by these mRNAs. Therefore, it is conceivable that the organization of these region could differ depending on dosage dependence of the proteins encoded. In this manuscript Wider et al explored how the features of the upstream mRNA regions vary in the genes with different tolerance to loss-of-function variants and phenotypical dosage dependence. The topic is interesting and important, I am not aware of similar studies. The result of the study mostly agree with what one may expect, it reaffirms the expectations for the association between dosage dependent genes and the presence of putative regulatory elements in the 5' regions of mRNAs, but also provide detailed characterization of this associatetion. Furthermore, the authors designed a viewer VuTR for visualization of 5' mRNA regions of human genes in the context of LOEUF score as a measure of loss-of-function tolerance and known variants in the upstream mRNA regions.

The overall impression is good, I am particularly pleased by the comprehensive description of the study limitations in the Discussion section. Nonetheless there are inaccuracies in the way how regulatory elements in the 5' regions are described, also some analyses could be improved to reduce the influence of confounding factors.

Suggestions for improving the analysis:

1. A major confounding factor is the level of gene expression. This is because the study relies on RiboSeq uORFs as detected with ribosome profiling (date form ref. 17). A major factor influencing detection of RiboSeq uORFs is coverage, therefore there is an expected correlation between the levels of gene expression and the presence of RiboSeq ORFs. To analyze the relationship between different groups of genes and the presence of uORFs, genes need to be stratified based on their expression. The same issue relates to CAGE analysis, the higher the gene expression the more likely multiple transcription sites would be identified simply due to elevated signal-to-noise ratio. So again, to strengthen the claim that LoF intolerant genes are enriched with multiple transcription initiation starts, they need to be stratified by gene expression levels.

2. Another confounding relationship is between GC content and the occurrence of AUG initiated uORFs. AUGs are less likely in GC rich regions by chance than in AT rich regions. The authors have shown that LoF intolerant genes have more AUGs but fewer uAUGs per base than the tolerant LoF genes. However, they also showed that LoF intolerant genes have higher GC content in the 5' regions. I expect a correlation between GC content and AUG occurrence simply due to the composition. One way to deal with this confounding factor is to reshuffle the sequences of 5' regions preserving the nucleotide composition and dinucleotide bias (another potential confounder linking RNA structure and AUG occurrence). If there is a genuine negative selection on uAUGs we would expect fewer uAUGs in the real 5' regions in comparison with the reshuffled 5' regions. Such analysis should strengthen the claim.

3. The authors noted "Despite this overall depletion, 52.1% of LoF intolerant genes (bottom quintile of LOEUF) contain at least one uAUG, suggesting that they may play an important role in translational regulation of these genes." But the authors correctly stated citing ref. 13 that about half of all human mRNAs contain uAUGs. Therefore, this number is pretty much what would be expected by chance. If these uAUGs were indeed regulatory, I would expect them to be more conserved across related species. The authors may try to analyze the conservation of uAUGs in order to support this statement, e.g. look at phylo-p scores exactly at AUG codons (rather than entire 5' regions).

Suggestions for improving the text:

4. The manuscript would benefit from a clear definition of terms. How ORF is defined? There are two common definitions of ORFs, start-to-stop and stop-to-stop (Sieber et al 2018 DOI: 10.1016/j.tig.2017.12.009). It seems to me that the authors use here the start-to-stop definition because they consider upstream in-frame starts extending CDS as starts of ORFs overlapping CDS (same stops, different starts). Many other researchers operate stop-to-stop ORF definition where two inframe starts with no inframe stops between them would be considered as belonging to the same ORF. To avoid potential confusion it would be helpful to give explicit definition of how ORF is defined in this work.

5. While the manuscript explains what are "genes intolerant to loss-of-function", it is unclear how disease genes are defined. The manuscript describes the sources of these gene sets, but for one outside of medical genomics, it would be useful to see a very brief explanation of how these gene sets were obtained in the first place.

6. UTRs stand for untranslated and the topic of translated ORFs is an apparent oxymoron, which is not atypical to molecular biology, we name things before we adequately understand them. Therefore, some researchers try to move away from the term and use mRNA leaders for 5' UTRs and mRNA trailers for 3' UTR. I fully understand that a suggestion to change the terminology for this specific manuscript may be impractical, but I encourage the authors to consider it in the future.

Background section

7. First paragraph. When describing regulatory elements in the 5' regions of mRNA the authors cite a review by Leppek et al (ref. 2). A substantial part of this review is dedicated to the claims of the existence of internal ribosome initiation sites in HOX genes. This claim was disproven independently by two labs (see Ivanov et al 2022 DOI: 10.1073/pnas.2117226119 and Akirtava et al doi: 10.1073/pnas.2122170119). Indeed looking at publicly available data there seems to be no evidence that the corresponding regions are ever become a part of mRNA. I suggest to use more reliable reviews on the same topic such as Hinnebusch et al 2016 DOI: 10.1126/science.aad9868, there are many other good and reliable reviews on the topic.

8. Third paragraph. It may be useful to mention a critical factor that determines translation initiation at the inspected start, namely the number of starts upstream of it. This factor correlates with the distance to the 5' end but is likely even more important since the number of preinitiation complexes is reduced after each subsequent start (Michel et al 2014 doi: 10.1186/s12859-014-0380-4).

9. Fourth paragraph. The current paradigm in annotation of human genes is to use the first AUG (within stop-to-stop ORF containing CDS) as a start of CDS unless there is explicit counter evidence. As a result, uAUGs inframe with CDS (depicted in Fig. 1a right bottom corner) are extremely rare and usually arise in the situations when the annotators of transcripts extend them upstream revealing such uAUGs while CDS annotations remain the same. Thus, most of extensions result from non-AUG initiation (Fedorova et al 2022 doi: 10.1038/s41467-022-35595-6). Perhaps it is worth acknowledging this, otherwise the impression is that there are many uAUGs inframe with CDS.

10. The same paragraph. The authors state that approximately half of the protein-coding genes contain at least one uORF citing Calvo et al (ref. 13). Calvo et al made the estimate based on the number of uORFs initiated only with AUGs. This estimate of uUAGs is correct, a more precise updated number of human mRNAs with uAUGs is available in Andreev et al 2022 doi: 10.1186/s13059-022-02674-2 for both Gencode and Refseq mRNA catalogs. However, given the large amount of non-AUG initiation, especially in the close proximity to 5' ends, the number of translated uORFs is likely to be much higher with the majority of mRNAs containing uORFs. Perhaps it is worth mentioning.

11. The same paragraph. At the end of this paragraph the authors make two statements that seem to be mutually exclusive, on one hand the uORF containing genes have lower expression (ref. 16) on the other hand uORFs positively correlate with translation of CDS (ref. 17-19). The authors provide no reconciliation to this contradiction even though it is apparent. Ref. 16 dealt with uORFs based purely on sequence, while Refs 17-19 are ribosome profiling studies that focused on detection of translated small ORFs (smORFs). A major factor in detection of translated smORFs is coverage, hence it is apparent that more translated smORFs would be detected in highly expressed genes, but this is simply a detection bias. This is because there is no evidence that we are even approaching saturation in detection of smORFs with increased coverage. The deeper the sequencing the more smORFs are identified, once the saturation is achieved the correlation observed in ref. 17-19 will likely disappear. Another issue is that mentioning refs 17-19 in such wording gives the impression that uORFs may enhance translation of CDS which is contrary to our understanding of translation initiation mechanisms and also contradicts what was observed in global analyses of how uORFs affect translation of CDS, see ref. 23 that is cited later in a different context.

12. Fifth paragraph. The authors state "Ribo-seq has shown that near-cognate codons (i.e those that differ from AUG by only a single base, such as CUG and GUG) can also act as functional uORF initiation sites [9,12]". Ribo-seq technique was first published in 2009, while ref. 9 is dated 2002 and ref. 12 is dated 2005. This must be corrected. Fortunately, there are plenty of literature on non-AUG initiation detection with ribo-seq, so it shouldn't be difficult to find something appropriate.

13. Same paragraph: "The efficiency of this ribosome reinitiation has been observed to decrease as the distance between the end of a uORF and start of the CDS decreases [22,23], as the scanning ribosomal subunit requires time (and therefore space) to reacquire a charged tRNAiMet with which to recognise the next start codon [9]." Reinitiation is complicated and the statement that its efficiency decreases with decreased distance to CDS is somewhat superficial description. Distance indeed plays a role, especially when it is very short, but the relationship is far more complicated and may not even be monotonous at longer distances. Regarding availability of tRNAiMet, this fact is relevant to the conditions of cellular stress when the availability of ternary complex complex (eIF2*GTP*tRNAi) drops significantly due to eIF2alpha phosphorylation. In this case indeed a long distance is required for post-termination scanning complex to acquire the ternery complex, while during normal conditions the ternary complex is readily available and reinitiation occurs at a short distance. This mechanism, known as "delayed reinitiation" is used to upregulate GCN4 expression in yeast during stress response. Obviously, the detailed account of reinitiation mechanism is out of the scope of the manuscript and a short sentence is better than many details. But I would advice the authors to word it carefully. For example one may say that "efficiency of reinitiation de is dependent on the length uORF, its sequence and its distance to CDS". Also while refs. 22 and 9 are appropriate here (both are reviews), ref. 23 may not be the best here since it is a research paper that does not explicitly deals with the relationship between reinitiation efficiency and the distance between uORF and CDS. Ref. 23 is more appropriate as evidence of inhibitory effect of uORFs on CDS translation.

Section "Genes intolerant to loss of function have longer and more complex 5'UTRs"

14. Third paragraph. Change "low minimum free energy (MFE)" to "low minimum free energy (MFE) of predicted secondary RNA structures"

Figures:

15. Since the manuscript deals not only with AUG but also with plenty of non-AUG initiated uORFs, I suggest using "start" instead of "AUG" in Fig. 1A. This is particularly relevant to in-frame oORF extensions since those are almost always non-AUG (see above).


Questions regarding VuTR:

16. VuTR is very nice and certainly useful especially when used in conjunction with other resources. However, there is a coupe of oddities that confused me. The uORFs as well as variants are often shown on different lines (variants seem to be always on two). These are clearly not the reading frames. What are they? Is their purpose to overcrowding of the displayed items or something else? When zooming to the sequence base leve, the dashes representing variants do not align perfectly to corresponding base letters. As a result, figuring out which nucleotide is effected isn't always easy. This is a minor but frustrating inconvenience. I also was wondering wouldn't it be helpful to represent variants as base letters, so that it is immediately clear what the variant is without a need to select it? Perhaps it isn't trivial to implement though. Another minor source of frustration is that the viewer does not allow to reverse the view onto the negative strand. I noticed many circles with "i" and "?" symbols which I was hoping would link to some help or additional information related to the current pages. However, clicking them didn't take me anywhere.

**Reviewer 2**

This manuscript provides a systematic overview of 50UTR regulatory elements and aims to find functional correlations with Loss of Function (LoF) propensity. Indeed, it shows that genes more sensitive to LoF have longer and more conserved 5'UTRs, which tend to be more structured and contain more uAUGs and uORFs with respect to more tolerant LoF genes.
The presented results are certainly of great interest for a large audience and open new avenues for functional validations and biotechnological applications.
However, I have some concerns which need to be addressed on the inferred statistical significance of investigated correlations.

1) Many 5'UTRs are incomplete or misannotated. The Authors should report statistics on a more restricted "golden" subset for which the completeness of 5'UTRs is supported by experimental evidence (e.g. CAGE signals).

2) The 5'UTR length increases with decreased tolerance to LoF (Fig. 2A) is not apparent. A clearer description of LOEUF deciles should be provided. In particular, for sake of clarity, I suggest to modify the legend on the x-axis from "LOEUF decile" to "tolerance to LoF". In addition, plotted figures should be reported on a supplementary Table (e.g. average and median, with SDs). This remarks applies to all plotted correlations in Fig.2.

3) The Authors should exclude an artifactual correlation between 5'UTR length and LOEUF, which could be simply due a different mRNA length. If this is the case any correlation should be found between LOEUF and CDS and 3'UTR length. In addition correlation data could be shown between the length ratio (5'UTR L / mRNA L) and LOEUF.

4) Concerning the possible correlation between secondary structure propensity and LOEUF, it is evident that again this could be artifactual due to the length bias previously mentioned. Indeed, a lower MFE is expected for longer 5'UTRs which tend to be more structured than shorter ones.

5) It is not clear how the PhyloP score has been calculated for the entire length of each UTR also because this could generate statistical biases (see also remarks above).

6) I suggest to provide a summary table for the different gene categories (DD, Inc, TSG, HS, TS, ..).

Minor remark

Fig.2C is menzioned before Fig. 2C

**Authors’ response to reviewers**

We would like to thank the reviewers for their positive comments on our manuscript and for their constructive suggestions. We have made changes to the manuscript and believe it is improved. Below is a full point-by-point response to each suggestion.

Reviewer 1

1. A major confounding factor is the level of gene expression. This is because the study relies on RiboSeq uORFs as detected with ribosome profiling (date form ref. 17). A major factor influencing detection of RiboSeq uORFs is coverage, therefore there is an expected correlation between the levels of gene expression and the presence of RiboSeq ORFs. To analyze the relationship between different groups of genes and the presence of uORFs, genes need to be stratified based on their expression. The same issue relates to CAGE analysis, the higher the gene expression the more likely multiple transcription sites would be identified simply due to elevated signal-to-noise ratio. So again, to strengthen the claim that LoF intolerant genes are enriched with multiple transcription initiation starts, they need to be stratified by gene expression levels.

We fully agree that the level of gene expression would impact discovery of Ribo-seq uORFs and transcription start sites using CAGE. To account for the confounding effect of gene expression, we repeated our analyses for both Ribo-seq uORFs and CAGE peaks across LOEUF deciles splitting genes into four quartiles based on their expression levels in GTEx. In each case, results remained significant in all four groups. We have included these results in Supplementary Figures 5 and 7 and have edited the main text and methods as below. Notably, our analysis of ‘predicted’ uORFs, based purely on the presence of AUGs within 5’UTRs would not be biassed in this way and shows consistent results with our Ribo-seq uORF analyses.

The main text now includes:
“This is true both using predicted uORFs and the uORFs detected by Ribo-Seq (Supplementary Figure 4A) and remains true when correcting for different gene expression levels which can impact detection of uORFs in Ribo-seq data (Supplementary Figure 5).” and
“As this analysis may be confounded by gene expression levels, with more highly expressed genes having more associated CAGE peaks, we repeated this analysis splitting genes into four quartiles of mean expression across tissues in GTEx. The result remained significant in all four quartiles (Supplementary Figure 7, all Chi-square P<8x10-11).

The methods now includes:
“We used gene expression data from GTEx (Genotype-Tissue Expression project) RNAseq based analysis file from GTEx called “Median gene-level TPM by tissue” (23 October 2023) [46]. GTEx collects and analyses gene expression levels from a wide range of tissue samples. We took a mean gene expression per gene across all tissues (measured in TPM - transcript per million). We split the data into 4 quartiles (Q1-Q4), ranging from low expression to high expression.”

2. Another confounding relationship is between GC content and the occurrence of AUG initiated uORFs. AUGs are less likely in GC rich regions by chance than in AT rich regions. The authors have shown that LoF intolerant genes have more AUGs but fewer uAUGs per base than the tolerant LoF genes. However, they also showed that LoF intolerant genes have higher GC content in the 5' regions. I expect a correlation between GC content and AUG occurrence simply due to the composition. One way to deal with this confounding factor is to reshuffle the sequences of 5' regions preserving the nucleotide composition and dinucleotide bias (another potential confounder linking RNA structure and AUG occurrence). If there is a genuine negative selection on uAUGs we would expect fewer uAUGs in the real 5' regions in comparison with the reshuffled 5' regions. Such analysis should strengthen the claim.

Again, we agree that GC content could bias our reported depletion of AUGs. To account for this, as suggested, we shuffled the sequence of each 5’UTR 1000 times while preserving the di-nucleotide composition. This analysis showed a strong depletion of AUGs compared to expected.

The following has been added to the main text: “To ensure that an overall depletion of uAUGs across 5’UTRs is not confounded by sequence composition (i.e. differences in GC content) we shuffled all MANE 5’UTR sequences 1000 times while maintaining di-nucleotide composition. AUGs were significantly more depleted than would be expected by chance (Supplementary Figure 6).” with an expansion of the associated methods included in the methods section.


3. The authors noted "Despite this overall depletion, 52.1% of LoF intolerant genes (bottom quintile of LOEUF) contain at least one uAUG, suggesting that they may play an important role in translational regulation of these genes." But the authors correctly stated citing ref. 13 that about half of all human mRNAs contain uAUGs. Therefore, this number is pretty much what would be expected by chance. If these uAUGs were indeed regulatory, I would expect them to be more conserved across related species. The authors may try to analyze the conservation of uAUGs in order to support this statement, e.g. look at phylo-p scores exactly at AUG codons (rather than entire 5' regions).

We agree that cross-species conservation is a good marker of the importance of regulatory uAUGs. This analysis is already included in Figure 2C where we separately present PhyloP scores at both uAUGs and uORF stop codons (in dark blue).

4. The manuscript would benefit from a clear definition of terms. How ORF is defined? There are two common definitions of ORFs, start-to-stop and stop-to-stop (Sieber et al 2018 DOI: 10.1016/j.tig.2017.12.009). It seems to me that the authors use here the start-to-stop definition because they consider upstream in-frame starts extending CDS as starts of ORFs overlapping CDS (same stops, different starts). Many other researchers operate stop-to-stop ORF definition where two inframe starts with no inframe stops between them would be considered as belonging to the same ORF. To avoid potential confusion it would be helpful to give explicit definition of how ORF is defined in this work.

We have expanded the details on how we define uORFs. Specifically, we have added the following text: “Where multiple uAUGs were in-frame to the same stop codon, all were considered as separate uORFs. Each uORF was therefore annotated as from the uAUG to the first in frame stop codon (i.e. using a start-stop ORF definition).”

5. While the manuscript explains what are "genes intolerant to loss-of-function", it is unclear how disease genes are defined. The manuscript describes the sources of these gene sets, but for one outside of medical genomics, it would be useful to see a very brief explanation of how these gene sets were obtained in the first place.

Thank you for alerting us to the fact that our explanations were insufficient for readers outside of the medical genomics field. We have now expanded our methods to include further details on each gene set.

6. UTRs stand for untranslated and the topic of translated ORFs is an apparent oxymoron, which is not atypical to molecular biology, we name things before we adequately understand them. Therefore, some researchers try to move away from the term and use mRNA leaders for 5' UTRs and mRNA trailers for 3' UTR. I fully understand that a suggestion to change the terminology for this specific manuscript may be impractical, but I encourage the authors to consider it in the future.

We fully agree that the term “untranslated region” is misleading. Due to complexities in altering the full manuscript, we decided to not change the terminology throughout, however, we have now mentioned this point in the discussion. Specifically, we have added: “Notably, 3’UTRs can also contain small translated regions (termed downstream ORFs, or dORFs). Hence, it may be more accurate to term 5’ and 3’ UTRs as ‘mRNA leaders’ and ‘mRNA trailers’, respectively, rather than using the term ‘untranslated’.” We will endeavour to change our terminology accordingly in future work.

7. First paragraph. When describing regulatory elements in the 5' regions of mRNA the authors cite a review by Leppek et al (ref. 2). A substantial part of this review is dedicated to the claims of the existence of internal ribosome initiation sites in HOX genes. This claim was disproven independently by two labs (see Ivanov et al 2022 DOI: 10.1073/pnas.2117226119 and Akirtava et al doi: 10.1073/pnas.2122170119). Indeed looking at publicly available data there seems to be no evidence that the corresponding regions are ever become a part of mRNA. I suggest to use more reliable reviews on the same topic such as Hinnebusch et al 2016 DOI: 10.1126/science.aad9868, there are many other good and reliable reviews on the topic.

Thank you, we have now included the suggested reference instead.

8. Third paragraph. It may be useful to mention a critical factor that determines translation initiation at the inspected start, namely the number of starts upstream of it. This factor correlates with the distance to the 5' end but is likely even more important since the number of preinitiation complexes is reduced after each subsequent start (Michel et al 2014 doi: 10.1186/s12859-014-0380-4).

We have now added this point, and the associated reference, to the background section of the manuscript.

9. Fourth paragraph. The current paradigm in annotation of human genes is to use the first AUG (within stop-to-stop ORF containing CDS) as a start of CDS unless there is explicit counter evidence. As a result, uAUGs inframe with CDS (depicted in Fig. 1a right bottom corner) are extremely rare and usually arise in the situations when the annotators of transcripts extend them upstream revealing such uAUGs while CDS annotations remain the same. Thus, most of extensions result from non-AUG initiation (Fedorova et al 2022 doi: 10.1038/s41467-022-35595-6). Perhaps it is worth acknowledging this, otherwise the impression is that there are many uAUGs inframe with CDS.

We do not believe that we give the impression that there are many uAUGs in-frame with the CDS. Indeed, we include the proportion of genes with in-frame uAUGs in our analysis (Figure 1B). There are many nuances of gene annotation that we do not go into detail on in our introduction given these are not the focus of our analysis and this would make it too long. Instead, we note differences in transcript annotation as a limitation of our work in the discussion.

10. The same paragraph. The authors state that approximately half of the protein-coding genes contain at least one uORF citing Calvo et al (ref. 13). Calvo et al made the estimate based on the number of uORFs initiated only with AUGs. This estimate of uUAGs is correct, a more precise updated number of human mRNAs with uAUGs is available in Andreev et al 2022 doi: 10.1186/s13059-022-02674-2 for both Gencode and Refseq mRNA catalogs. However, given the large amount of non-AUG initiation, especially in the close proximity to 5' ends, the number of translated uORFs is likely to be much higher with the majority of mRNAs containing uORFs. Perhaps it is worth mentioning.

The reference from Calvo et al. is used within this paragraph as our narrative here is focussed on uAUG initiation. We have instead added the suggested reference to support an additional sentence in the following paragraph where we introduce Ribo-seq and the concept of non-AUG initiation. Specifically, we have added “Considering these non-AUG start codons dramatically increases the number of potentially translated upstream start codons.”.

11. The same paragraph. At the end of this paragraph the authors make two statements that seem to be mutually exclusive, on one hand the uORF containing genes have lower expression (ref. 16) on the other hand uORFs positively correlate with translation of CDS (ref. 17-19). The authors provide no reconciliation to this contradiction even though it is apparent. Ref. 16 dealt with uORFs based purely on sequence, while Refs 17-19 are ribosome profiling studies that focused on detection of translated small ORFs (smORFs). A major factor in detection of translated smORFs is coverage, hence it is apparent that more translated smORFs would be detected in highly expressed genes, but this is simply a detection bias. This is because there is no evidence that we are even approaching saturation in detection of smORFs with increased coverage. The deeper the sequencing the more smORFs are identified, once the saturation is achieved the correlation observed in ref. 17-19 will likely disappear. Another issue is that mentioning refs 17-19 in such wording gives the impression that uORFs may enhance translation of CDS which is contrary to our understanding of translation initiation mechanisms and also contradicts what was observed in global analyses of how uORFs affect translation of CDS, see ref. 23 that is cited later in a different context.

We agree that these recent results as presented are confusing and contradictory. We have made two changes to aid clarity. Firstly, we have removed the second statement on positive correlation from its original location in the manuscript and have altered the text in this location to more clearly state that uORFs generally down-regulate CDS translation. Specifically, this section now reads “It is estimated that half of all protein-coding genes contain at least one uORF [13], and that they generally result in a decrease in translation of the downstream protein. Indeed, active uORF translation has been observed to reduce downstream translation by up to 80% [13]. Genes with uORFs have been demonstrated to have lower protein expression levels than genes without uORFs in multiple human tissues [16].”

Secondly, we have moved the note of the observed “positive correlation” to the end of the next paragraph, which seems more appropriate given it pertains to ribo-seq data. While we do not provide a reconciliation given it seems this is unresolved in the literature (and a long discussion is out of scope here), we do now present this with more doubt. Specifically, we include “Recent Ribo-seq studies have suggested that uORF translation is generally positively regulated with translation of the CDS [18–20], however, this is at odds with uORFs that have been fully characterised [13,17] and it is currently unclear if this reflects biology or is an artefact of Ribo-seq.”.

12. Fifth paragraph. The authors state "Ribo-seq has shown that near-cognate codons (i.e those that differ from AUG by only a single base, such as CUG and GUG) can also act as functional uORF initiation sites [9,12]". Ribo-seq technique was first published in 2009, while ref. 9 is dated 2002 and ref. 12 is dated 2005. This must be corrected. Fortunately, there are plenty of literature on non-AUG initiation detection with ribo-seq, so it shouldn't be difficult to find something appropriate.

Thank you for alerting us to this error in our referencing. We have changed the reference to the Ribo-seq dataset we use in the paper (Chothani et al. 2022 10.1016/j.molcel.2022.06.023) as this clearly demonstrates this point.

13. Same paragraph: "The efficiency of this ribosome reinitiation has been observed to decrease as the distance between the end of a uORF and start of the CDS decreases [22,23], as the scanning ribosomal subunit requires time (and therefore space) to reacquire a charged tRNAiMet with which to recognise the next start codon [9]." Reinitiation is complicated and the statement that its efficiency decreases with decreased distance to CDS is somewhat superficial description. Distance indeed plays a role, especially when it is very short, but the relationship is far more complicated and may not even be monotonous at longer distances. Regarding availability of tRNAiMet, this fact is relevant to the conditions of cellular stress when the availability of ternary complex complex (eIF2*GTP*tRNAi) drops significantly due to eIF2alpha phosphorylation. In this case indeed a long distance is required for post-termination scanning complex to acquire the ternery complex, while during normal conditions the ternary complex is readily available and reinitiation occurs at a short distance. This mechanism, known as "delayed reinitiation" is used to upregulate GCN4 expression in yeast during stress response. Obviously, the detailed account of reinitiation mechanism is out of the scope of the manuscript and a short sentence is better than many details. But I would advice the authors to word it carefully. For example one may say that "efficiency of reinitiation de is dependent on the length uORF, its sequence and its distance to CDS". Also while refs. 22 and 9 are appropriate here (both are reviews), ref. 23 may not be the best here since it is a research paper that does not explicitly deals with the relationship between reinitiation efficiency and the distance between uORF and CDS. Ref. 23 is more appropriate as evidence of inhibitory effect of uORFs on CDS translation.

Thank you again for ensuring the accuracy of the wording in our introduction. We have removed the original reference 23 from this point in the text and have altered the sentence to read: “The efficiency of this ribosome reinitiation has been observed to be dependent on the length of the uORF, its sequence, and the distance between the end of a uORF and start of the CDS [10, 23].”

Section "Genes intolerant to loss of function have longer and more complex 5'UTRs"

14. Third paragraph. Change "low minimum free energy (MFE)" to "low minimum free energy (MFE) of predicted secondary RNA structures"

We have made this suggested edit. The sentence now reads “The propensity of a sequence to form RNA secondary structures can be predicted from high GC content and low minimum free energy (MFE) of predicted secondary RNA structures”.

15. Since the manuscript deals not only with AUG but also with plenty of non-AUG initiated uORFs, I suggest using "start" instead of "AUG" in Fig. 1A. This is particularly relevant to in-frame oORF extensions since those are almost always non-AUG (see above).

Thank you for this suggestion. We agree, and have changed figure 1A to say start codon instead of AUG.

16. VuTR is very nice and certainly useful especially when used in conjunction with other resources. However, there is a coupe of oddities that confused me. The uORFs as well as variants are often shown on different lines (variants seem to be always on two). These are clearly not the reading frames. What are they? Is their purpose to overcrowding of the displayed items or something else? When zooming to the sequence base leve, the dashes representing variants do not align perfectly to corresponding base letters. As a result, figuring out which nucleotide is effected isn't always easy. This is a minor but frustrating inconvenience. I also was wondering wouldn't it be helpful to represent variants as base letters, so that it is immediately clear what the variant is without a need to select it? Perhaps it isn't trivial to implement though. Another minor source of frustration is that the viewer does not allow to reverse the view onto the negative strand. I noticed many circles with "i" and "?" symbols which I was hoping would link to some help or additional information related to the current pages. However, clicking them didn't take me anywhere.

Thank you very much for your positive comments and constructive feedback on VuTR. We have implemented the following changes in response:
● We have added base letters within the variant tracks such that users can easily view which base a certain variant is changed to.
● Alignment to the sequence has been fixed. Although we note that this isn’t always perfect when using the zoom feature due to our use of the package visualisation.
● We have fixed the bug that was causing the Help text to not show up when clicking on the appropriate buttons.

Regarding the visualisation of multiple uORFs, there aren’t any well supported and maintained JavaScript packages specifically pertaining to transcript-native visualisations and building something custom off the shelf is non-trivial. Within the constraints of existing packages, we need to display the uORFs on separate tracks when they overlap. We are looking into alternative custom visualisations for future updates as we aim to continually update the application based on user feedback.

Reviewer 2

1) Many 5'UTRs are incomplete or misannotated. The Authors should report statistics on a more restricted "golden" subset for which the completeness of 5'UTRs is supported by experimental evidence (e.g. CAGE signals).

Throughout our analysis we used a transcript set defined by the MANE project. This is an effort jointly run by NCBI and Ensembl to define a high quality set of transcripts. The project assigns a single high-confidence transcript per gene based on a host of available data including using CAGE data to define 5’UTRs. Hence, all of our statistics are reported on a set of transcripts which are supported by experimental data. We have extended the section of the methods where we describe the transcript definition to detail that these are CAGE supported. Specifically it now reads: “We used MANE Select transcripts from v1.0 of the MANE resource [27] to define a single 5’UTR per gene. Of 19,062 MANE Select transcripts, 18,764 had annotated 5’UTRs. Notable, CAGE data from the FANTOM5 project was used by MANE to inform 5’UTR definition.”

2) The 5'UTR length increases with decreased tolerance to LoF (Fig. 2A) is not apparent. A clearer description of LOEUF deciles should be provided. In particular, for sake of clarity, I suggest to modify the legend on the x-axis from "LOEUF decile" to "tolerance to LoF". In addition, plotted figures should be reported on a supplementary Table (e.g. average and median, with SDs). This remarks applies to all plotted correlations in Fig.2.

Thank you for alerting us to the fact that the correlation between LOEUF scores and tolerance to LoF in Figure 2 was unclear. While we do not believe it is appropriate to change the label on the x-axis (as this should detail exactly what is being plotted), we have added an arrow at the bottom of the figure to demonstrate how the gene tolerance to LoF varies across each plot.

Further, we have now reported all data plotted in figures 2 and 3 in Supplementary tables 1 to 7.

3) The Authors should exclude an artifactual correlation between 5'UTR length and LOEUF, which could be simply due a different mRNA length. If this is the case any correlation should be found between LOEUF and CDS and 3'UTR length. In addition correlation data could be shown between the length ratio (5'UTR L / mRNA L) and LOEUF.

We agree that it would be good to include this analysis. Hence, we have now added a Supplementary Figure showing (5’UTR length / total mRNA length) by LOEUF decile and the following text to paper: “Further, we find that the proportion of the total mRNA that is annotated as 5’UTR is significantly greater for genes in the lowest LOEUF quintile compared to the highest quintile (Supplementary Figure 2B; Wilcoxon Rank Sum, P<1x10-15) indicating that LoF intolerant genes have longer 5’UTRs even after accounting for the total length of the mRNA.”.

4) Concerning the possible correlation between secondary structure propensity and LOEUF, it is evident that again this could be artifactual due to the length bias previously mentioned. Indeed, a lower MFE is expected for longer 5'UTRs which tend to be more structured than shorter ones.

We agree that this is an important bias to exclude and have included an MFE analysis in the manuscript where we restricted it to 5'UTRs between 100 and 300 bps long.

5) It is not clear how the PhyloP score has been calculated for the entire length of each UTR also because this could generate statistical biases (see also remarks above).

We have added more detail to the methods section on how we calculated the mean PhyloP scores. Specifically, it now reads: “5’UTR bases were further annotated with per-base vertebrate PhyloP scores (phyloP100way) retrieved in R using the GenomicScores package. Separate means were calculated for each gene across (1) all 5’UTR bases, (2) all uORF start and stop codons within the 5’UTR, and (3) all bases of start-stops within the 5’UTR.” Given that we calculate mean PhyloP scores across each base of each 5’UTR rather than using for e.g. a maximum or minimum value, this should not be overly biassed by length.

6) I suggest to provide a summary table for the different gene categories (DD, Inc, TSG, HS, TS, ..).

As noted in response to your point (2), we have now included these data in Supplementary Table 7.

7) Fig.2C is menzioned before Fig. 2C

We assume you meant that Fig 2C is mentioned before Fig 2B. Thank you for pointing this out. We have now changed the order of panels in the figure so they are mentioned in order.

**Second round of review**

**Reviewer 2**

The Authors adequately addressed all the remarks.
